# Progress in Surgery

**Published:** 1895-09-07

**Authors:** 


### Medical Progress and Hospital Clinics.

[*The Editor will be glad to receive offers of co-operation and contributions from members of the profession. All letters should be addressed to* The Editor, The Lodge, Porchester Square, London, W.]

### Progress in Surgery.

### ORTHOPÆDIC SURGERY.

**Congenital Deformity of the Chest.**—Dr. Irving S. Haynes[1] contributes an interesting article with illustrative cases. He commences by stating that the congenital deformities of the chest may be referred to the sternum, ribs, or cartilages, and may involve any one or all three factors. In the sternum the abnormalities may be complete cleft of the sternum, and of this he quotes a case of Dr. Sinclair's recorded in the *Dublin Journal of Medical Sciences*, 1887, p. 557, partial cleft, or the persistence of one or more holes through the bone. The congenital variation in the ribs consists of extra cervical or lumbar ribs, deficiencies in the normal ribs, or fusion of two ribs. Of these deformities examples are quoted.

**Supports in Spinal Disease.**—Dr. A. M. Phelps[2] discusses the respective values of plaster of Paris, wood and aluminium, and other spinal supports. Regarding the plaster of Paris corset, he says that, after fourteen years of experience, it is one of the best supports in Pott's disease the world has ever seen; but it is apt to be heavy, and the fact that it is non-porous makes it objectionable. He has therefore substituted the wooden corset, the paper corset, and the aluminium corset. These combine all the good elements of the plaster of Paris. With reference to the aluminium corset, Dr. Phelps says that it has these qualities to recommend it to the patient—lightness, durability, and that it is so thin as not to interfere with the form of the patient; being extensively perforated it is the coolest and most agreeable of supports. An ordinary aluminium corset weighs from one to two pounds, and is covered with a waterproof enamel which protects it from perspiration. In shaping it, it is necessary to make a plaster of Paris model of the patient's trunk.

A vindication of the treatment of spondylitis and scoliosis by means of plaster of Paris bandages is proffered by Dr. Lewis A. Sayre.[3] After detailing his method of applying it, Dr. Sayre is at pains to relate fully the opinions of numerous orthopædists to whom he has written on the subjects. Opinions are given from all parts of the world, and it would seem that the plaster of Paris corset, properly applied, most nearly fulfils all the required indications.

Dr. J. C. Schapps[4] discusses the mechanical treatment of Pott's disease of the spine in the sub-acute or convalescent stage. He advocates a modification of the well-known Taylor's brace, and he claims that by his arrangement (1) all the available space is utilised for the application of force, both of leverage and traction; (2) the leverage is expended entirely in the antero-posterior direction; (3) the anterior and posterior braces nearly meeting at the sides, lateral support is amply provided in every case where recumbency is not needed; (4) the respiratory movements are not interfered with; (5) the back or any part of the trunk can be at any time readily inspected; (6) the apparatus is inexpensive.

**Scoliosis.**—W. F. Somerville[5] has recently visited the Central Institute of Gymnastics at Stockholm, and describes the methods practised there for the treatment of weak back, and adds that this treatment, rightly employed in suitable cases, is of the greatest service. The writer was also much impressed with the Zander method of treatment of contraction and deformities.

A. H. Tubby[6] has described the clinical aspects of scoliosis. He insists upon the distinctions between simple lateral deviation and scoliosis, rotation being the prominent factor in the latter; and he further gives a table of differences between the two affections. The clinical aspects of scoliosis are discussed under the following headings: (1) Cases in which the curvature is mainly unilateral, or C curves. (2) Cases of double, or S curve. (3) Cases in which three or more curves exist. (4) Cases of scoliosis associated with prominence of several spinous processes.

In a clinical lecture on "Lateral Curvature of the Spine," by Mr. Christopher Heath,[7] the view, first propounded by Mr. Barwell, that the over-action of the right seratus magnus is the determining factor in the production of dorsal curves to that side, is endorsed by the lecturer as correct. The system of treating all cases of lateral curvature of the spine by supports is rightly inveighed against. It is further advocated that the habits of the patient be inquired into, the legs be measured to see if any shortening is present, and that properly regulated exercises be enforced. It is also shown that some cases without osseous deformity, especially in weakly children, require support, and that the pain in cases of osseous deformity is frequently relieved by some form of apparatus.

Lateral decubitus as a cause and as a method of treatment of lateral curvature of the spine is fully entered into by Dr. N. Wilbur,[8] but we cannot agree with him that "it is only within recent years that lateral decubitus has been recognised as a cause of lateral curvature. If inquiry be made I presume that a large portion of the cases will be found to arise from this cause." It seems to us that the importance of lateral decubitus as an exaggerating factor in scoliosis has received full recognition of late years, and it is very doubtful if it is the cause of many of them, in our opinion.

A. H. Tubby[9] discusses the prognosis of lateral curvature of the spine from the following points of view—the cause, the age of onset, the sex, and the condition of the bodily health, the probable rate of growth of patient, the occupation, and the site and form of curve. It is stated that if curvatures commence in puberty they are more difficult to treat than those beginning after eighteen; that the worst cases arise in patients with long yielding spines; that lumbar curves are less favourable for treatment than dorsal; and that cervical curves are particularly troublesome to deal with, owing to the great effect of the weight of the head in increasing any existing curve.

**The Pathological Anatomy of Scoliosis.** — Hoffa[10]

dwells upon the great importance of the condition of the ascending articular processes. On the concave side they are considerably lower than on the convex, and the superior part of the joint is reduced to a thin transparent leaf of bone. The superior articular processes of the concave side are widened considerably in a lateral direction, so that they often involve the entire width of the articular processes. The vertical measurement of the bodies on the convex side is found to be greater than on the concave side, so that a wedge-shaped appearance results.

**Congenital Displacement of the Hip.**—Schede[11] has, since 1880, treated these cases by orthopædic methods, which have given him excellent results. His method rests upon the fact that in the vast majority of children with congenital hip dislocation, in whom no secondary changes have been caused by walking, a simple traction upon the leg and a slight abduction suffice to place the head in the acetabulum, and that further a moderate lateral pressure upon the great trochanter is only necessary to retain the head of the bone in this position. After the child has begun to walk, the prognosis is less favourable. Schede has thus reported his experience with twenty-nine cases: Four cured, eleven almost cured, and fourteen but slightly improved.

**Paralytic Club-foot.**—Dr. Winkelmann,[12] recognising that when the patient begins to walk without an apparatus the foot tends to assume more and more the position of talipes equino-varus, has devised a means by which the preponderating action of the plantar flexors and supinators can be thrown on to the pronators. The "operation" which he has devised for this purpose has only been performed once, in the case of a little girl aged six, and the result was excellent, and has been fully maintained This operation is better than that of amputation of both legs at the knee-joint for the same affection, which was performed by W. J. Taylor, of Philadelphia,[13] (although in the latter case the deformity was stated to have been of such a character, and the resulting disability so great, that no form of appliance or conservative operative proceeding appeared likely to give the desired results. Under such conditions Dr. Taylor was of the opinion that amputation, followed by properly fitting and accurately adjusted artificial limbs, was the only means of positively benefiting the patient.

**Treatment of Congenital Club-foot.**—W. Barton Hopkins[14] has practised removal of about half-an-inch of the lower end of the fibula for inveterate talipes varus with success. He argues that the deformity produced by Potts' fracture, in which the fibula is frequently shortened, is the exact reverse of that seen in talipes varus.

Mr. Woollcombe,[15] writing on club-foot, carefully discusses the various theories of the origin of club-foot, both congenital and acquired, and in the case of the former believes that the great majority are due to disordered nervous action. The article is a careful *résumé* of the various opinions held by authorities.

**Flat-foot.**—Mr. Openshaw[16] discusses the vexed question of the origin of acquired flat-foot. He states that the chief factor is prolonged standing on both feet with the body slightly bent forwards and with the feet turned out, since this is the position in which most weight is thrown upon the transverse metatarsal arch. Von Meyer has pointed out that when standing upon both feet with the body slightly bent forwards, the body-weight presses upon the centre of the internal plantar arch. Mr. Openshaw advocates various forms of exercises, together with manipulation and an appropriate support. Sir William Stokes[17] also discusses in full the origin of flat-foot. He bases his arguments on the incidence of the body-weight in various positions upon the inner line of Von Meyer's triangle, and shows the effect of that pressure in the production of flat-foot. Ogston's ingenious operation, whereby firm osseous ankylosis between the astragalus and scaphoid is produced is well spoken of by Sir William, but he suggests a modification of it consisting of an astragaloid osteotomy removing the greater part of the head of the bone, forcibly adducting the foot, refreshing the astragaloid surface of the scaphoid and fixing the two bones together, thereby raising the arch permanently and rendering the weak spot immovable.

**Morton's Disease or Metatarsalgia.** — Goldthwait[18] thinks that the cause of this affection is obliteration of the transverse metatarsal arch, and that the pain is due to the formation of a certain amount of inflammatory callus around the head of the metatarsal bones, arising from injury or fall.

[1] Amer. Medico. Surg. Bulletin, Nov. 15, 1894, p. 1356. [2] New York Med. Jour., Mar., 1895, p. 387. [3] New York Med. Jour., Mar., 1895, p. 332. [4] New York Med. Jour., Mar. 23, 1895. [5] Glasgow Med. Jour., Nov., 1894. [6] The Hospital, June 1, 1895, p. 147. [7] Brit. Med. Jour., Mar. 16, 1895, p. 573. [8] New York Med. Jour., Dec. 8, 1894, p. 794. [9] Clinical Journal, Dec. 5, 1894, p. 90. [10] Annals of Surgery, Dec., 1894. [11] Annals of Surgery, Mar., 1895. [12] Medical Week, April 5, 1895. [13] Therapeutic Gazette, Dec. 15, 1894, p. 822. [14] Annals of Surgery, April, 1895. [15] The Hospital, March, 9, 1895, p. 401. [16] Clinical Journal, Dec. 12, 1894, p. 106. [17] Brit. Med. Jour., Dec. 1, 1894, p. 1224. [18] Amer. Med. Surg., Bulletin, Nov., 1894.

